# Census data aggregation decisions can affect population‐level inference in heterogeneous populations

**DOI:** 10.1002/ece3.6475

**Published:** 2020-06-25

**Authors:** Søs Engbo, James C. Bull, Luca Börger, Thomas B. Stringell, Kate Lock, Lisa Morgan, Owen R. Jones

**Affiliations:** ^1^ Department of Biology University of Southern Denmark Odense Denmark; ^2^ Department of Biosciences Swansea University Swansea UK; ^3^ Natural Resources Wales Bangor UK; ^4^ Natural Resources Wales Martin's Haven UK; ^5^ RSPB Ramsey Island St Davids UK; ^6^ Interdisciplinary Center on Population Dynamics (CPOP) University of Southern Denmark Odense Denmark

**Keywords:** conservation, grey seal, matrix population modeling, population dynamics, population management, survey methods

## Abstract

1. Conservation and population management decisions often rely on population models parameterized using census data. However, the sampling regime, precision, sample size, and methods used to collect census data are usually heterogeneous in time and space. Decisions about how to derive population‐wide estimates from this patchwork of data are complicated and may bias estimated population dynamics, with important implications for subsequent management decisions.
2. Here, we explore the impact of site selection and data aggregation decisions on pup survival estimates, and downstream estimates derived from parameterized matrix population models (MPMs), using a long‐term dataset on grey seal (*Halichoerus grypus*) pup survival from southwestern Wales. The spatiotemporal and methodological heterogeneity of the data are fairly typical for ecological census data and it is, therefore, a good model to address this topic.
3. Data were collected from 46 sampling locations (sites) over 25 years, and we explore the impact of data handling decisions by varying how years and sampling locations are combined to parameterize pup survival in population‐level MPMs. We focus on pup survival because abundant high‐quality data are available on this developmental stage.
4. We found that survival probability was highly variable with most variation being at the site level, and poorly correlated among sampling sites. This variation could generate marked differences in predicted population dynamics depending on sampling strategy. The sample size required for a confident survival estimate also varied markedly geographically.
5. We conclude that for populations with highly variable vital rates among sub‐populations, site selection and data aggregation methods are important. In particular, including peripheral or less frequently used areas can introduce substantial variation into population estimates. This is likely to be context‐dependent, but these choices, including the use of appropriate weights when summarizing across sampling areas, should be explored to ensure that management actions are successful.

## Graphical abstract

Population management planning often uses models parameterised from survey data. These data are often aggregated across time and space. We illustrate how data aggregation decisions can influence inferences and highlight that these choices could have important consequences for future management.

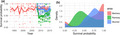

## 1 INTRODUCTION

As human impact on ecosystems increases (Goudie, 2013; Halpern et al., 2008; Newbold et al., 2016; Pullin, 2002) so does the need for tools that enable us to effectively undertake and assess conservation and management initiatives. Estimates of vital rates (e.g., survival and fecundity) are vital for management planning. Matrix population models (MPMs), which describe the age/stage‐based life cycle of a population at a particular time and place, are an important tool to this end (Ezard et al., 2010). The elements of MPMs describe a population's demography by quantifying survival probabilities, rate of ontogenetic development from stage‐to‐stage (or age to age), and reproduction. These MPMs can be used to project future population size and structure by multiplying them with an initial population vector, and a variety of population and life history metrics can be derived from them (Caswell, 2001).

Demography deals with the structure and dynamics of populations, and hence, the characteristics of populations, for example, size, fecundity, and survival (Caswell, 2001; Sodhi, 2010). It is these characteristics that help us describe populations and enable us to act if a population is on the verge of extinction or has become invasive. However, the raw census data that are used to parameterize MPMs and other models are often heterogeneous in both quality and quantity, often varying both spatially and temporally, which is highly likely to affect all estimates derived from these models—including the predicted future population dynamics. This heterogeneity in the data can have an important impact on population viability analyses (PVAs) and conservation management (Morris & Doak, 2002). Consequently, decisions taken during the early stages of the analysis, when researchers make estimates of vital rates, are crucial. Therefore, knowledge about how data handling affects the inferences drawn from models is much needed. To ensure that conservation efforts (irrespective of the species) are fruitful, focus on methodology is of the essence.

### 1.1 The importance of methodology

Gray seals were eradicated due to hunting from most European waters in the 16th and 17th century and around Britain the population declined to between 2,000 and 4,000 individuals in the beginning of the 20th century (Härkönen et al., 2007; Summers, 1978). Conservation efforts since then have ensured that populations are now viable and cover large parts of the species' historic range (Fietz et al., 2016). The conservation success (Lambert, 2002; Thomas, 2009) and resulting recent rise in abundance have led to renewed interest in human–wildlife conflicts. For example, seals have been blamed for declining commercial fish stocks and damage to equipment (Bosetti & Pearce, 2003; Butler, Middlemas, Graham, & Harris, 2011; Cook, Holmes, & Fryer, 2015; Moore, 2003). Therefore, there is a need for reliable data and predictions of population size to inform wildlife management. To be able to assess such issues, valid predictions of population dynamics of the gray seal must be modeled. MPMs and other models are useful for this, but are highly dependent on the raw census data they are based upon, which can be heterogeneous in quantity and quality. Moreover, the census data represent samples from a spatial process. This means that decisions on how the census data are collected and handled potentially influence the conclusions that can be drawn from subsequent population models. An additional challenge is that the collection of census data is time consuming and requires specially trained personnel. It is therefore imperative that the idiosyncrasies of the data are well understood, and that they are collected as efficiently as possible, to maximize the quality of inferences derived from the data. We note that these issues are not unique to seals, and apply widely to many animal species.

**FIGURE 1:**
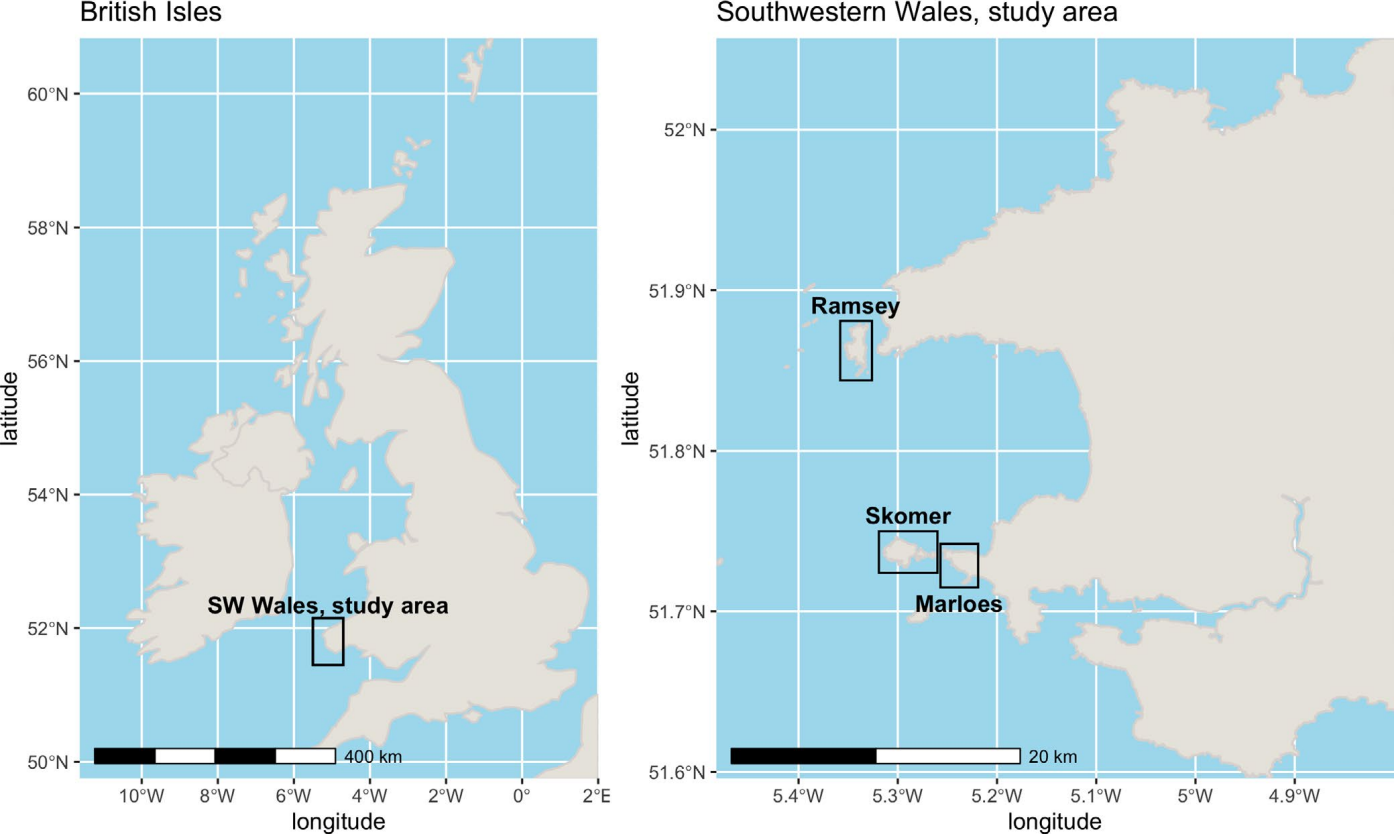
The location of the three main sampling areas of Ramsey Island, Skomer Island and the Marloes Peninsula in southwestern Wales, UK. Within each sampling area, there are numerous sampling sites (beaches and caves), with 9 on Ramsey, 19 on Skomer and 18 on Marloes (See Figure S1 in Supp Info for detailed maps)

In this paper, we examine how different methods summarizing the raw data affect derived estimates of population dynamics. The rationale behind this is to address the fact that large‐scale long‐term monitoring data are often collected using heterogeneous methods across heterogeneous sub‐populations that experience different environmental conditions, and in cases when the ease of survey varies. For example, some sites may be monitored frequently, others less so; some sites may be monitored remotely (e.g., with binoculars/telescope), while others may be more closely monitored; some sites may permit the marking of individuals while others may not. The drivers of this variation include ease of access, time considerations, and population size or “desirability” of the site. We explore how estimates of seal pup survival probability vary over 25 years across 46 specific sampling sites within three main sampling areas of southwestern Wales (Figure 1), and how decisions about how the data are combined may influence inferences. We combine these pup survival estimates with juvenile and adult survival probabilities derived from the literature to create a set of MPMs which we use to explore how site selection and data aggregation decisions could influence predicted population dynamics. We find that spatial variation among beaches within the three large sampling areas (rather than year‐to‐year temporal variation) dominates, and that this variation in survival estimates could have potentially large effects on derived population dynamics estimates, depending on how the data are used in population models. Our results highlight the importance of understanding spatial and temporal variation when conducting demographic studies of widespread species.

## 2 MATERIALS AND METHODS

### 2.1 Estimates of pup survival

Gray seal pups in Pembrokeshire, Wales, have been counted and monitored for several years by Natural Resources Wales (NRW) on Skomer Island (292 ha, 51.738°N 5.297°W) and Marloes Peninsula (51.732°N 5.234°W) (Lock, Newman, Burton, & Jones, 2017), and by the Royal Society for the Protection of Birds (RSPB) on Ramsey Island (260 ha, 51.865°N 5.339°W) (Morgan, 2014), hereafter referred to as Skomer, Marloes, and Ramsey. These locations fall within the Pembrokeshire Marine Special Area of Conservation, designated under the European Habitats Directive (Council Directive 92/42/EEC) and Pembrokeshire Coast National Park. Marloes and Skomer are part of the Skomer Marine Conservation Zone, and Ramsey Island is a National Nature Reserve. In this paper, we use a subset of these data collected from 1993 to 2014 on Marloes, from 2012 to 2015 on Skomer, and from 2008 to 2015 on Ramsey (Figure 1, Figure S1).

Forty‐six specific sampling sites (beaches and caves; hereafter, simply sites) across the three main sampling areas (Skomer, Marloes and Ramsey) have been monitored several times per week during the breeding season (August 1st–November 30th). The monitoring method differed among the three sampling areas. On Skomer, there are 19 sites and daily surveys focused on the six sites where the largest number of pups are born, while the remaining sites were surveyed every fourth day. On all of these surveys, pups on the accessible beaches were dye‐marked with colored aerosol sheep‐fleece marker sprays to provide a unique identity number indicated by particular color combinations (Büche & Stubbings, 2016). On a part of Marloes (~7 km of coastline), the surveys were carried out every 2–4 days at 18 sites. However, the frequency of visits depended on whether pups were present or not—locations with more pups were visited more frequently. In this sampling, area pups were not dye‐marked and, instead, wardens compared observations with the previous visit's record and subsequently estimated if/how many of the pups had survived to the next age/stage. On Ramsey, surveys were carried out every third day at 9 sites. Pups were not dye‐marked and all surveys were done from cliff tops—similar to the method used at Marloes. Due to the large size of some of the sites and seal populations on Ramsey, the Ramsey survey method required an adjustment factor to correct for the probability of double counting neonate pups on consecutive surveys (Strong, Lerwill, Morris, & Stringell, 2006). This correction factor results in noninteger estimates of pup numbers and sometimes resulted in more survivors than the initial pup number. For our analysis, we rounded these values to the nearest integer and, in the 9 out of 72 cases for which the number of survivors was greater than the initial number of pups, we assumed that estimated number of survivors is the correct value and that the probability of survival has been 1.

During data collection, pups were classified at all locations using a method based on Radford, Summers, and Young (1978) which classifies pups into five developmental stages based on physical features and approximate age. We assumed that pups reaching “Stage IV,” which is characterized by a rounded barrel‐shaped body with no visible neck and the beginning of fur molting, survived until independence. This stage is reached after approximately 16–20 days, after which the pup is independent of the mother (Radford et al., 1978).

### 2.2 Exploring the impact of data source and aggregation choices

The survey methods resulted in 450 estimates of pup survival, spread across the 46 sites within the three main sampling areas over several years. We carried out two sets of analyses using these data: In the first set, we examined variation in survival across time and across sites within the three sampling areas described above, and in the second set, we explored the potential impact of this variation and sampling strategy on estimates of population dynamics.

We estimated the average survival probability for each of the three sampling areas by calculating the arithmetic weighted mean across all sites in each sampling area every year, with weights defined by the number of seals at each site. We then examined these three time‐series of survival probability to assess differences among sampling areas and years. We tested for a directional trend using linear models regressing the weighted mean against year. In addition, to examine variation in survival in the three sampling areas, we plotted the density distribution of the site‐year‐specific survival probabilities. This plot provides an indication of how much influence the broad “sampling area” can have on estimated survival probability, and how much variation in survival exists within each broad sampling area (i.e., across sites and years). Furthermore, we estimated the percentage of total explained variance that was due to sampling area, site, or year effects using hierarchical partitioning of variance (Chevan & Sutherland, 1991) implemented using the *hier.part* R package (Mac Nally & Walsh, 2004).

To reveal the degree to which particular sites tend to pull up, or drag down, the overall mean we subsetted the data to years for which all three sampling areas are represented and carried out a sensitivity analysis in which we systematically recalculated the overall mean pup survival after removing each site's data one‐by‐one. We then examined whether these sensitivity estimates were associated with the seal density by modeling the association between sensitivity and the mean number of pups observed at each site (which is very highly correlated with the number of adult females). This relationship is potentially important because surveys tend to be carried out more intensively at more populous sites, which could introduce bias.

In addition to this sensitivity analysis, we investigated how many site‐and‐year‐specific estimates of survival are required to obtain an adequate estimate of the overall mean survival. To do this, we randomly sampled from the survival estimates from sites with varying sample sizes from 5 to 50 and calculated the mean from this sample. We repeated this sampling procedure 1,000 times and calculated the proportion of times that the mean of the sample was within 10% of the overall mean obtained from using all available data. We followed this procedure for all three sampling areas lumped together, and within each of the three sampling areas separately.

**FIGURE 2:**
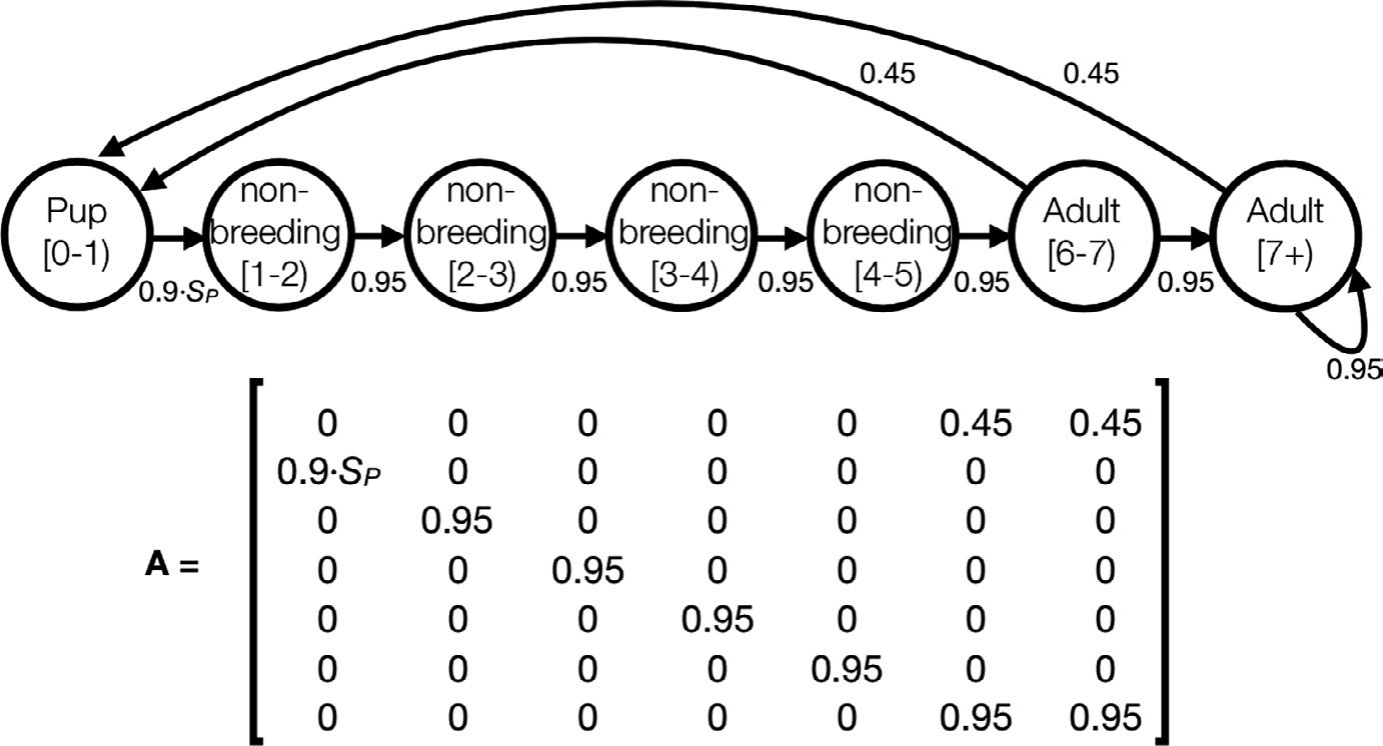
The matrix model we used for this study based on Thomas et al. (2019). Pup survival (*S* *_P_*) was estimated as the critical early‐stage survival (*S_E_* from birth to “Stage IV” *sensu* Radford et al. (1978) multiplied by 0.7 to account for late‐stage mortality (*S_L_*) from pup Stage IV to 1 year of age. See main text for details). The fecundity estimate of 0.9 was multiplied by the sex ratio at birth of 0.5 to ensure a female‐based model

We used our pup survival estimates to parameterize MPMs by combining them with the estimates made in a recently published MPM of UK gray seal vital rates (Thomas et al., 2019). Like Thomas et al. (2019), our MPM (Figure 2) has seven stages: pups aged 0–1 years; prebreeding females (age classes 1–5 years); and breeding‐age females (6+ years). Survival probability for both prebreeding and breeding‐age females was estimated to be 0.95 and fecundity was estimated to be 0.90 (Table 1 in Thomas et al. (2019)). Thomas et al. (2019) estimated maximum pup survival from age 0 to 1 year (*S*_0‐1_), at low population density, to be 0.48, but in our models, we replaced this value with pup survival estimates calculated from our own data. Our pup survival estimates (*S_E_*) are for the early, most critical, period of life (from day 0 to Radford et al. (1978)'s “Stage IV,” which is reached after approximately 20 days). To estimate survival from 0 to 1 year, we assumed that survival probability after passing the critical period (after Stage IV, to 1 year of age, denoted *S_L_*) was constant and that 0‐to‐1‐year‐survival (S_0‐1_) could be estimated as *S_E_*⋅ *S_L_*. Therefore, we could estimate *S_L_* as 0.48/*S_E_* = *S_L_*. Like Thomas et al. (2019), we assumed a 50:50 sex ratio at birth, and we therefore multiplied fecundity by 0.5 to model only the female part of the population. We make the implicit assumption that the adult population is well‐mixed and that there are therefore no differences in transition probabilities for adult stages.

Based on this MPM archetype, we then constructed four sets of MPMs: (a) 46 MPMs based on the across‐year weighted mean pup survival at each site (18 for Marloes, 9 for Ramsey, 19 for Skomer), (b) three MPMs parameterized using the sampling area‐specific weighted mean survival probability, (c) two MPMs parameterized using the weighted mean survival probability of the ten consistently worst‐ and best‐performing sites, and (d) twenty MPMs parameterized using the weighted mean of the ten worst‐ and ten best‐performing sites (in terms of survival probability). In all cases, the weights were defined by the seal number of seal pups at each site. We thus constructed a total of 74 MPMs, each with a different pup survival probability. We only varied pup survival among sites in these MPMs and assumed that other transition rates are fixed. It is certainly possible that other transition rates do vary spatially, but we argue that there will be little difference in at‐sea distribution, so we think this is a reasonable assumption. In addition, we emphasize that the purpose of these models is not to create accurate population projections per se but rather to investigate the effect of variation and uncertainty in estimates of pup survival, which is likely to be the most variable transition rate.

We estimated population growth rate (*λ*) for each of these MPMs and examined the result graphically by plotting the smoothed density distribution of the site mean *λ* estimates. To this plot, we added the three *λ* estimates for MPMs constructed using the sampling area means for Marloes, Ramsey and Skomer, and the two *λ* estimates from the MPMs constructed using data from the sites with lowest and highest survival probabilities. This plot gives a good indication of the potential bias introduced when using estimates derived from the three sampling areas, and depending on typical survival probabilities at the different sites.

We examined potential variation in the fate of the populations using stochastic projection methods (Morris & Doak, 2002) to project populations with an initial population structure of 575 pups, 1,724 sub‐adults (divided evenly among the four non‐breeding stages), and 576 adults (divided equally between the two breeding stages). These initial pup and adult counts were taken from Morgan (2014) and Büche and Stubbings (2016), and we assumed that there would be around three times as many juveniles based on the population structures reported in these publications. We noted, however, that the initial values are not particularly important due to the asymptotic nature of the projections. Briefly, the stochastic projection works by randomly selecting one of a set of MPMs for each year of the projection. To examine the effect of long‐term average differences in survival among sites, we used MPMs generated using early‐stage pup survival estimates (*S_E_*) from all sites. We subsetted these matrices to produce two sets from the ten worst‐ and ten best‐performing sites (with performance defined by the long‐term site‐specific average survival probability). Each of these two sets included MPMs for each site, parameterized for each year of available data, to ensure that both temporal variation was represented in the projections. We projected the initial population vector stochastically for twenty years to assess population size and long‐term stochastic population growth rate.

## 3 RESULTS

**FIGURE 3:**
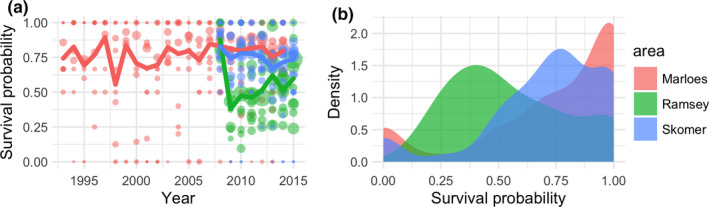
(a) Pup survival probability varied through time in the three main sampling areas, Marloes (red), Ramsey (green), and Skomer (blue). The lines show mean survival in each sampling area. Points represent survival for a particular site in a given year, color coded by sampling area and with point sizes proportional to number of pups observed. (b) The density distribution of site‐specific survival probabilities (including all years) shows that survival differs among the main sampling areas

We analyzed how the source of data affects both pup survival estimates and estimated population growth rates and population projections using long‐term data collected at Marloes, Skomer, and Ramsey. Pup survival varied through time and depends on the sampling area from which the data are collected (Figure 3a,b). Although fewer years of data are available for Ramsey and Skomer, it is apparent that survival probabilities tend to be lower for pups born at sites in these main sampling areas. Survival tended to be highest in Marloes and lowest in Ramsey (Figure 3b), but the probability distributions show that choice of site within these sampling areas is also crucial, since pup survival shows great variation among these sites, as indicated by the distribution of individual data points. There was no directional trend in survival probability (OLS regression: for Marloes *F*_1,20_ = 1.716, *p* = .205; for Ramsey *F*_1,6_ = 0.144, *p* = .718; for Skomer *F*_1,6_ = 5.497, *p* = .057).

**FIGURE 4:**
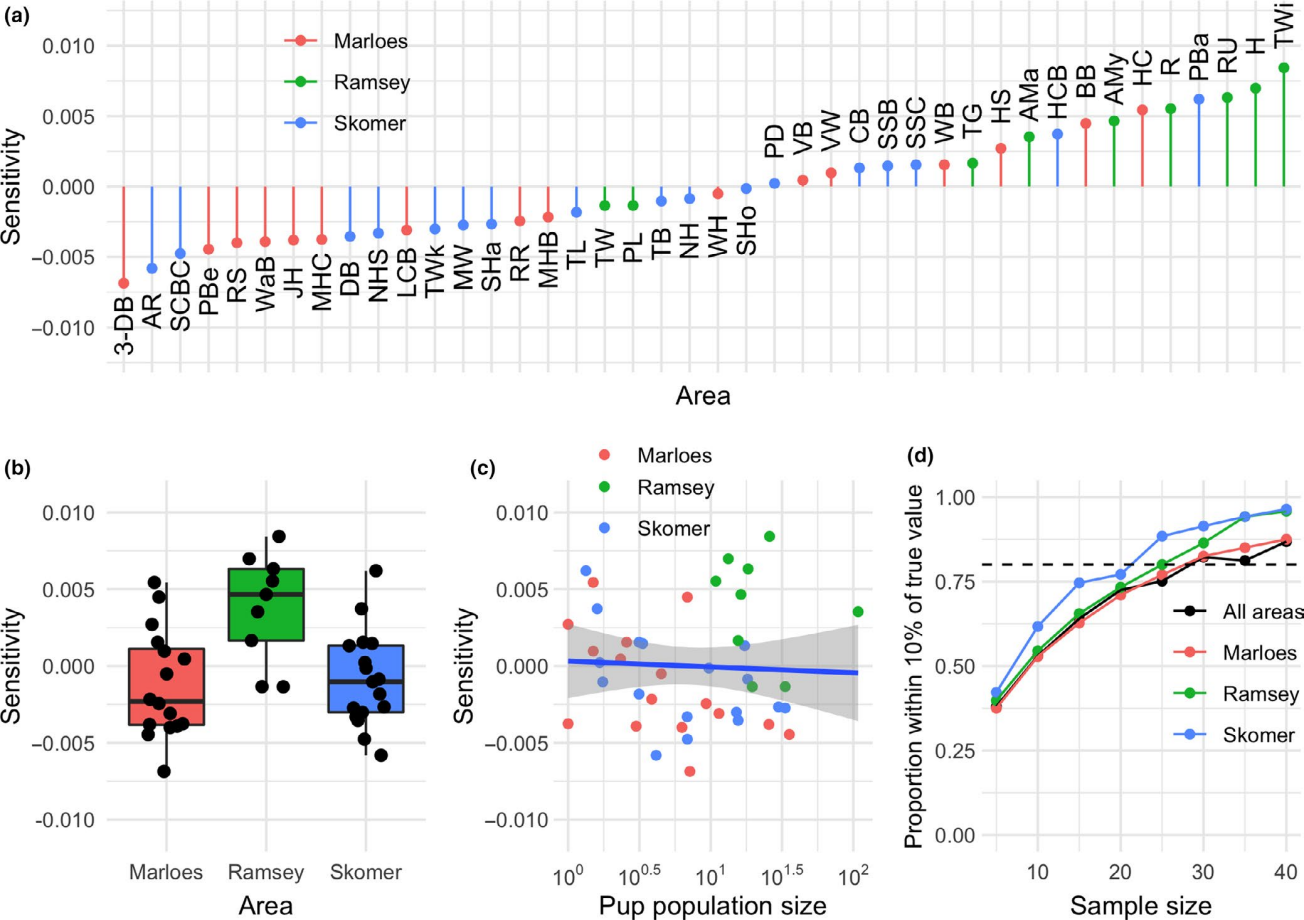
(a) Sensitivities of the overall survival estimate to omission of particular sites. Positive values indicate that omitting the site leads to an increased survival estimate. The overall mean pup survival probability is 0.69, so a sensitivity of 0.01 indicates that omitting the site would increase the mean estimate to 0.70. See Supplementary Material for key to the site codes. (b) Sensitivities vary by sampling area, with sensitivities for Ramsey sites tending to be higher than those of Marloes and Skomer. (c) There is no statistically significant association between sensitivity and population size, indicating that favoring high‐population sites should not bias survival estimates. (d) The number of samples (site and/or year) required to achieve an estimate of within 10% of the best estimate, using all data with a probability of 0.80 (indicated with the dashed line), varied among the main sampling areas between ~20 (for Skomer) and ~30 (for Marloes)

Our sensitivity analysis revealed that exclusion of particular sites can pull up or down the overall mean survival estimate of 0.69 by between approximately −0.01 and 0.01 (i.e., an increase/decrease of ~1.45%) (Figure 4a). Sensitivities varied among sampling areas, with Ramsey having higher sensitivity than Skomer or Marloes (i.e., omission of points from Ramsey tended to pull up overall survival estimates) (linear model: *F* = 14.0565 on 2 and 38 *df*, *p*‐value < 0.001; Figure 4b). There was no statistically significant association between the sensitivity and site population size (linear model: *F* = 0.1648 on 1 and 38 *df*, *p*‐value = 0.6871) (Figure 4c). Our assessment of sample size requirements to achieve a good estimate of the mean survival (Figure 4d) showed that to achieve an estimate that fell within ± 10% of the best estimate (i.e., using all data for the sampling area) varied among the three main sampling areas. Sample size requirements, i.e. the number of survival estimates drawn from specific sites in specific years, ranged from ~20 for Skomer to ~30 for Marloes.

From the overall mean survival estimate of 0.69, and assuming that survival from birth to 1 year (*S_0‐1_*) is approximately the same as reported by Thomas et al. (2019) (0.48), we estimated that the late‐stage survival from Stage IV to 1 year (*S_L_*) is approximately 0.70 (i.e., 0.48/0.69). We used this *S_L_* estimate in the matrix models (below) to calculate survival from birth to age 1 (*S*_0‐1_) from *S_E_* as described above.

**FIGURE 5:**
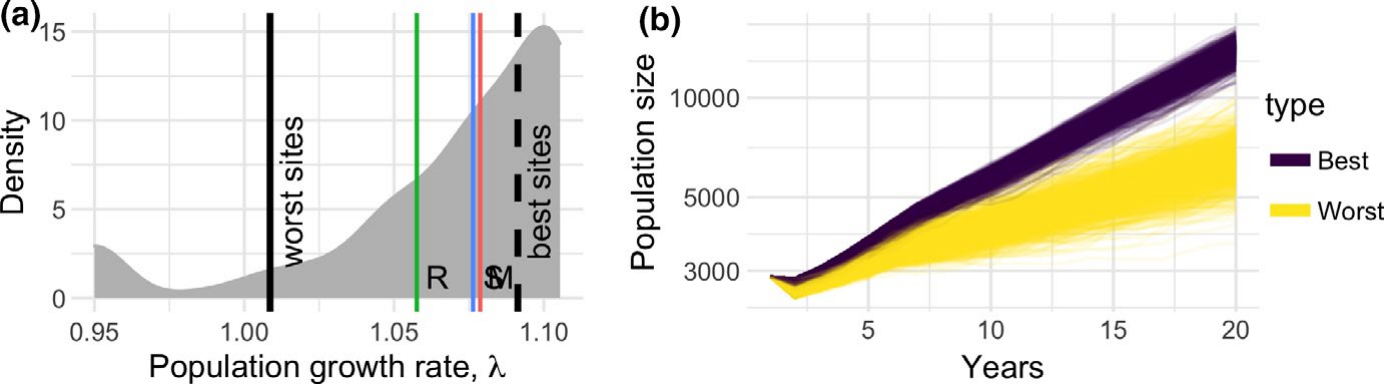
(a) Density distribution of population growth rates (gray) calculated from matrix projection models (MPMs), in which pup survival was estimated from each site. Vertical lines indicate population growth rates estimated from MPMs using mean pup survival for each main sampling area (marked R, S, and M for Ramsey, Skomer, and Marloes, respectively), and for MPMs using mean pup survival from the ten sites with the worst and best pup survival. (b) The consequences of data source for projections of population development, showing 1,000 stochastic projections of population size using MPMs where pup survival estimates were taken from the ten sites with the worst and best survival. Initial population structure was 575 pups, 1,724 sub‐adults (divided evenly among the four nonbreeding stages) and 576 adults (divided equally between the two breeding stages). To enable visualization of the transient phase, only the first 20 years of the projection are shown. Note that the vertical axis is presented on a log scale

Our investigation of how pup survival estimates influence the derived estimates from matrix population models (MPMs) showed that the source of the data was critical. Population growth rates estimated from MPMs using pup survival estimates derived from different sites showed variation from 0.95 to 1.11 (i.e., a population decrease of 5% per year to a population increase of 11% per year) (Figure 5a). Use of the mean pup survival estimates from sites in the three main sampling areas produced *λ* estimates of 1.06, 1.08, and 1.09, respectively, for Ramsey, Skomer, and Marloes (i.e. 6%, 8%, and 9% increases per year). When pup survival estimates were derived from the ten worst‐, and ten best‐performing sites (in terms of pup survival), λ was estimated to be 1.04 and 1.09, respectively (i.e., a 4% and 9% increase). This variation has consequences for population size projections (Figure 5b), with markedly different projected population growth depending on whether the best or worst sites were used for stochastic population projections.

**FIGURE 6:**
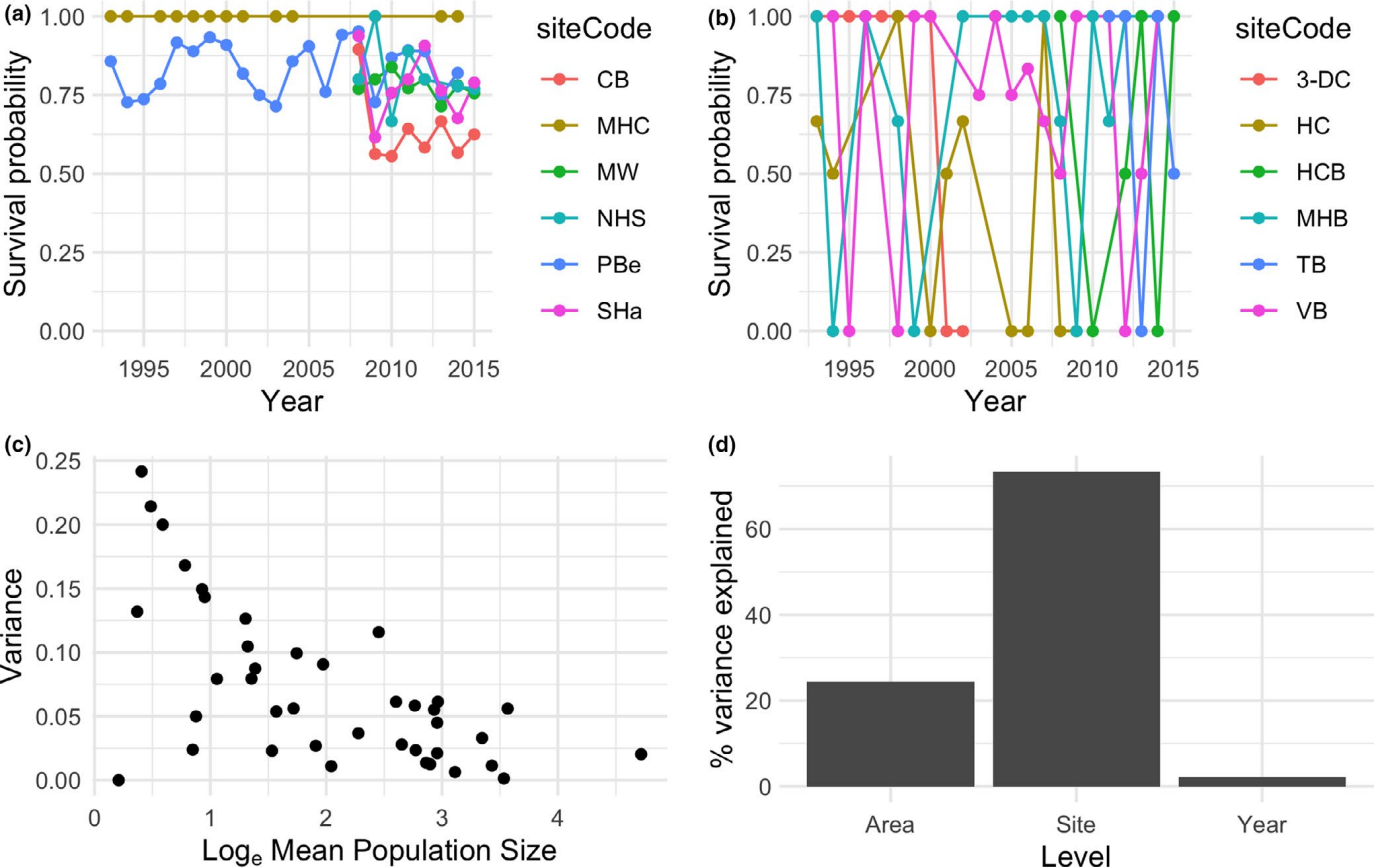
The temporal variation in survival probability differed markedly among sites within the three main sampling areas (Skomer, Marloes, and Ramsey), with some showing high variance and others showing low variance. As an example, panel (a) shows the trajectories of the six lowest‐variance sites and panel (b) shows the trajectories of the six highest‐variance sites. Temporal variance tends to decline with average pup population size (c). Hierarchical partitioning (d) of this variance indicates that most variation in survival (74%) is due to site‐level differences, with 24% of variation due to sampling area effects and only 2% due to year effects. The site names indicated in panels A are as follows: Castle Bay (CB), Martin's Haven Cave (MHC), Matthew's Wick (MW), North Haven Slip (NHS), Pebbly Beach (PBe), South Haven (SHa). The site names in panel B are as follows: 3‐Doors Cave (3‐DC), Horseshoe Cave (HC), High Cliff Boulders (HCB), Martin's Haven Beach (MHB), The Basin (TB) and Victoria Bay (VB)

There was a large amount of variation among sites (Figure 5a), but we also found trends within these data. The year‐to‐year variance in survival probability differed markedly among sites, with some sites showing relatively little variation (Figure 6a) compared with others (Figure 6b). These differences are largely caused by differences in pup population size estimates among sites (Figure 6c), with some sites attracting many seals while others attract only few. The formal hierarchical partitioning of variance showed that most variation in pup survival (74%) was due to site effects, while 24% was due to sampling area effects and only 2% was due to year effects (Figure 6d). We emphasize, however, that these results should not be taken to mean that there is little temporal variation—in fact, there is substantial temporal variation as shown in Figure 6a,b, but this temporal variation is site‐specific: temporal variation in survival is not well‐correlated among sites, as indicated by weak pairwise correlations (mean Pearson pairwise correlation coefficient for pairs with at least 5 data points = 0.113 ± 0.01 *SEM*; mean *p*‐value = .591 ± .01 *SEM*).

## 4 DISCUSSION

Monitoring wildlife and changes in the environment helps us understand, predict, and respond to changes in population dynamics (Gauthier, Péron, Lebreton, Grenier, & van Oudenhove, 2016). Without accurate information, we may fail to notice changes, such as those caused by human impact, which could have serious consequences for ecosystem function. In this study, we have investigated how monitoring methods (in terms of site selection) and data aggregation decisions may influence estimates of seal pup survival and the outputs of matrix population models derived from them. We show that there is great spatial and temporal heterogeneity in pup survival rates and that this heterogeneity has important consequences when aggregating these data to parameterize population models. Sensitivity analysis showed that omission of particular sites can lead to a modest change in estimated mean pup survival (~1.45%), and mean sensitivity varied among sites (Figure 4a,b). In addition, the sample sizes requirements to achieve a reasonably confident estimate of survival varied markedly among sampling areas (Figure 4d). Thus, the way in which the data are collected and/or aggregated can strongly influence the vital rate estimates, and consequently, the MPM‐derived metrics of population dynamics and life history traits. Differences in projections may not necessarily be important in the short‐term, but becomes critical in the longer term, as small effects are iteratively compounded. The fact that we found clear differences in survival among sites and in different sampling areas, driven by both site‐specific variation in monitoring method and environmental variation, mean that the sampling decisions are crucial when assessing and predicting population development.

Survival probability is affected by environmental factors in other pinniped species (Chambellant, Stirling, Gough, & Ferguson, 2012; McMahon, Harcourt, Burton, Daniel, & Hindell, 2017; Retana, Guzmán, & Lewis, 2013; Sundqvist, Harkonen, Svensson, & Harding, 2012). We found marked spatiotemporal variation in survival for the gray seal pups of southwestern Wales, but most of this variation is among sites rather than among the three larger sampling areas or among years. This among‐site variation in survival probability is likely to be driven by environmental factors such as the topography and size of the sites (Twiss, Duck, & Pomeroy, 2003), exposure to wind and waves, and perhaps food availability (Smout, King, & Pomeroy, 2010), but this is unlikely in this case due to the large‐scale movements of adults during the foraging season. The abundance of seals at the breeding locations might influence survival via density‐dependent processes, which may vary among sites. In addition, it is certain that stochasticity plays a major role, as evidenced by the relationship between site‐level temporal variation and average pup population size (Figure 4c). Collectively, these factors emphasize the importance of sampling area, understanding the source of the survival variation. Therefore, further work is needed to better understand the relative importance of these potential drivers. Our results emphasize the desirability of monitoring multiple sites that represent the spatial variation in site characteristics and survival probabilities.

Our analyses illustrate that the projected fate of the population can be skewed by inadvertently monitoring nonrepresentative sites, for example, only high‐ or low‐survival sites (Figure 5b). Planning the future management of the species based on estimates derived from nonrepresentative monitoring could lead to spurious inferences (e.g., of population stasis or decline when it is actually increasing). These errors could have serious consequences for future management. For example, they could lead to the initiation of unnecessary and/or costly actions, or to a false sense of security and failure to act.

Assessing the uncertainties in the predictions of future population dynamics is crucial for making informed management decisions (Mouquet et al., 2015). Our analysis suggests that decisions on how to collect and handle the gray seal pup census data can have large impacts on inferences. The relatively small scale spatial variation in demography we illustrate for the Welsh population also occurs at a larger spatial scale, as described in the yearly reports of the Natural Environment Research Council (NERC) Special Committee on Seals (SCOS). These reports make it apparent that population density and pup production vary considerably across the UK: The estimated pup production in Wales (1,650 pups) is a small fraction of the UK total (65,000 pups, 2016 estimate), which is dominated by the large (54,750 pups, 2016 estimate) contribution made by the Scottish population (SCOS, 2018). In addition, although there has been a ~5% increase in pup production UK‐wide between 2014 and 2016, this varied from +0.2% in Orkney to +18.3% in the Farne islands (SCOS, 2018). Although the Welsh contribution to the UK‐wide assessment is certainly relatively small compared with that of Scotland, the details, including temporal variation, are uncertain due to data limitations. In fact, the pup production estimate of 1,650, which has been used in every report since 2009, is based on surveys at indicator sites in 2004 and 2005, and on broader surveys in 1994. Thus, it is currently impossible to examine the UK data for changes in the Welsh contribution to the whole. Nevertheless, we urge caution in making extrapolations based on outputs from analyses conducted on limited population data when the population itself is widespread. This is evidently the case for the gray seal, and also undoubtedly applicable to numerous other species.

## CONFLICT OF INTEREST

The authors declare no competing interests.

## AUTHOR CONTRIBUTIONS

**Søs Engbo:** Conceptualization (supporting); formal analysis (supporting); funding acquisition (supporting); methodology (supporting); visualization (supporting); writing–original draft (lead); writing–review and editing (supporting). **James C. Bull:** Conceptualization (equal); data curation (equal); funding acquisition (supporting); investigation (supporting); methodology (supporting); supervision (supporting); writing–original draft (supporting); writing–review and editing (supporting). **Luca Börger:** Conceptualization (equal); data curation (equal); funding acquisition (supporting); investigation (equal); methodology (equal); supervision (supporting); writing–review and editing (supporting). **Tom B. Stringell:** Data curation (equal); funding acquisition (equal); investigation (supporting); methodology (supporting); project administration (equal); resources (equal); writing–review and editing (supporting). **Kate Lock:** Data curation (supporting); investigation (equal); methodology (equal); project administration (equal); writing–review and editing (supporting). **Lisa Morgan:** Data curation (supporting); investigation (equal); methodology (equal); project administration (equal); writing–review and editing (supporting). **Owen R. Jones:** Conceptualization (equal); formal analysis (lead); funding acquisition (supporting); investigation (equal); methodology (equal); project administration (supporting); software (lead); supervision (lead); visualization (lead); writing–original draft (supporting); writing–review and editing (lead).

### OPEN RESEARCH BADGES

This article has been awarded Open Materials, Open Data, Badges. All materials and data are publicly accessible via the Open Science Framework at https://doi.org/10.5281/zenodo.3385204.

## Supporting information

Supplementary Material

## Data Availability

Data and code enabling replication of this study are available at the following https://doi.org/10.5281/zenodo.3385204. Contains Natural Resources Wales information © Natural Resources Wales and Database Right. All rights Reserved.
