# Supplementary material for: Census data aggregation decisions can affect population‐level inference in heterogeneous populations
Source: Ecol Evol. 2020 Jun 25;10(14):7487–96. doi: 10.1002/ece3.6475 (PMC7391327; doi:10.1002/ece3.6475)
Supplement: Supplementary file 1 — Supplementary Material [file ECE3-10-7487-s001.pdf]

Supporting Information for Søs Engbo et al. "Census data aggregation decisions can affect population-level inference in heterogeneous populations".

Site locations on the main sampling areas

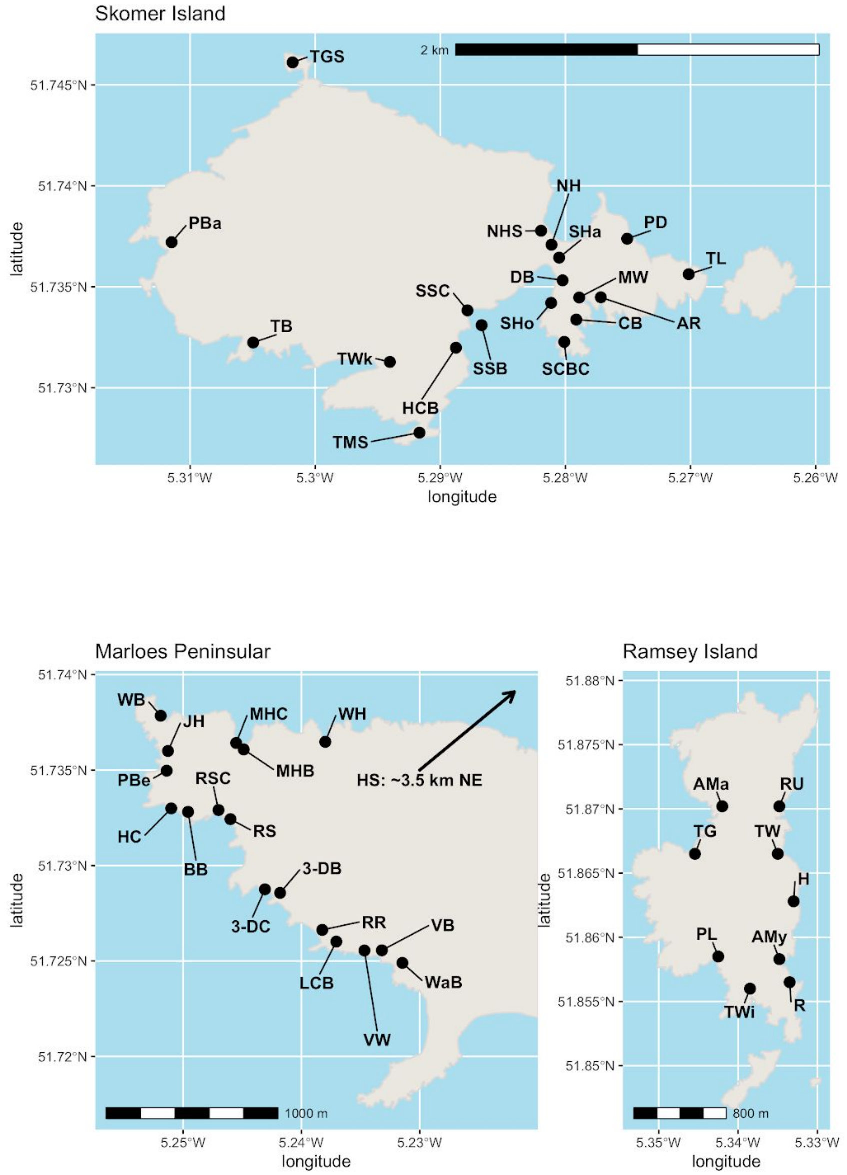

Figure S1: Site locations at the three main sampling areas in southwestern Wales. The site codes within each area are as follows. **Skomer Island (19 sites):** Amy's Reach (AR), Castle Bay (CB), Driftwood Bay (DB), High Cliff Boulders (HCB), Matthew's Wick (MW), North Haven (NH), North Haven Slip (NHS), Pigstone Bay (PBa), Protheroe's Dock (PD), Seal Hole (SHo), South Castle Beach Cave (SCBC), South Haven (SHa), South Stream Boulders (SSB), South Stream Cave (SSC), The Basin (TB), The Garland Stone (TGS), The Lantern (TL), The Mew Stone (TMS), The Wick (TWk). **Marloe Peninsular (18 sites):** 3-Doors Beach (3-DB), 3-Doors Cave (3-DC), Boulder Beach (BB), Horseshoe Cave (HC), Howney Stone (HS), Jeffery's Haven (JH), Little Castle Bay (LCB), Martin's Haven Beach (MHB), Martin's Haven Cave (MHC), Pebbly Beach (PBe), Rainy Rock (RR), Renny Slip (RS), Renny Slip Cave (RSC), Victoria Bay (VB), Victoria West (VW), Watery Bay (WaB), West Hook (WH), Wooltack Bay (WB). **Ramsey Island (9 sites):** Aber Mawr (AMa), Aber Myharan (AMy), Hwrddod (H), Porth Lleuog (PL), Rhod Uchaf (RU), Rhosyn (R), The Waterings (TW), Thomas Williams (TWi), Trwyn Garlic (TG).
